# New insights into mechanisms of material ejection in MALDI mass spectrometry for a wide range of spot sizes

**DOI:** 10.1038/s41598-018-25946-z

**Published:** 2018-05-17

**Authors:** Marcel Niehaus, Jens Soltwisch

**Affiliations:** 1 Institute for Hygiene, University of Münster, Robert-Koch-Straße 41, 48149 Münster, Germany; 2 Interdisciplinary Center for Clinical Research (IZKF), University of Münster, Domagkstraße 3, 48149 Münster, Germany

## Abstract

Matrix-assisted laser desorption/ionization mass spectrometry (MALDI-MS) is widely used for the analysis of large biomolecules in numerous applications. The technique utilizes nanosecond-long laser pulses at various spot sizes to eject and ionize large molecules embedded in a highly absorptive chemical matrix. Despite the methods name, ‘molecular desorption’ from the matrix crystal surface is not the sole mechanism discussed for material ejection in MALDI, but additional ablation of larger clusters has been reported. Here we present results on the influence of laser fluence and spot size on the mechanisms of the initial material ejection in MALDI and subsequent plume development. We used a laser-based postionization (MALDI-2) as well as a complementary photoacoustic method to monitor the material ejection step. The photoacoustic data reveal a quasi-thermal sublimation process up to a transition fluence. Above this threshold fluence additional ablation processes are observed. Complementary investigations on plume dynamics by MALDI-2 showed an ejection of predominantly fast particles for desorption conditions while ablation produces considerably slower ejecta. Additionally the presented results revealed a peculiar influence of the spot size on analyte fragmentation as well as plume development and allows for new insights into the unexplained spot size effect reported for MALDI.

## Introduction

Matrix-assisted laser desorption/ionization (MALDI) is a major technique for the mass spectrometric (MS) analysis of large, thermally labile biomolecules such as peptides/proteins, phospho- and glycolipids, or oligosaccharides^1^. In recent years it has gained great relevance in the field of imaging mass spectrometry^2–4^. Mechanistically, MALDI is based on a highly complex cascade of events triggered by rapid energy uptake that is delivered by a nanosecond-long laser pulse, usually in the near UV. The laser irradiation triggers highly convoluted processes of energy relaxation and distribution, co-release of analyte and matrix molecules from analyte-doped matrix crystals into the gas-phase, as well as primary and secondary ionization steps that finally lead to the production of intact analyte ions^5–7^. Over the course of 20 some years a multitude of fundamental studies has deepened our understanding of these processes. It is generally agreed and experimentally well verified that the sensitivity of a MALDI-MS analysis is critically determined by the physicochemical properties of the analyte/matrix system (such as optical absorptivity, proton affinities, and molar analyte-matrix ratios), on the one hand, and by a proper choice of irradiation parameters (such as laser wavelength, pulse duration, fluence, and focal spot size), on the other^8–15^. The mechanisms behind these empirical findings however often remain only poorly understood. While particularly the ionization step continues to be the topic of a lively discussion in the community^16–20^, also the initial step of material ejection is not fully comprehended. While a number of experimental results point to a molecular evaporation/sublimation from the surface^8,21,22^, also material ejection from the bulk volume including clusters and particulates has been reported^22–28^. Both mechanisms are described to lead to distinctively different physical features of the ejected plume. In the quasi-thermal model, material ejection is driven by a molecular sublimation process caused by an increased temperature on the heated surface. In the process often referred to as thermal desorption, matrix molecules rapidly detach from the upper layers and incorporated analyte is entrained. Beyond a threshold temperature this leads to largely complete phase transition from solid to gaseous of the topmost layers. Close to threshold fluence the process produces thermal molecules with velocities of up to 1000 m/s^5,29^. At elevated laser fluences increased particle densities may lead to the formation of a molecular jet^30^. The model predicts that internal energy of ejected particles is directly dependent on the surface temperature reached due to the laser irradiation and relaxation processes^31^.

The commonly used term ablation on the other hand describes an explosive disruption of a larger volume induced by rapid overheating^22,32^. In this model, the energy deposition rate by the laser pulse exceeds the consumption by all dissipation pathways (e.g. melting, sublimation, energy transport) and enables a supercritical heating. Subsequently tensile stress in the sample can nucleate different ablation processes leading to the disintegration of the bulk^33^. This leads to the ejection of single molecules but also of larger clusters of material, consisting of hundreds to thousands of molecules that may disintegrate further at a later stage in the plume. According to calculations and simulations by Zhigilei *et al*. these massive clusters can be assumed to have much lower particle velocities^34^.

A great obstacle of experimentally confirming the proposed mechanisms when using MALDI-ions as a probe lies in the strong entanglement of material ejection and ionization efficiency. Circumventing this problem, laser postionization (PI)^8,35,36^, laser-induced fluorescence imaging^27^, optical imaging^23^, and photoacoustic analysis (PA) experiments^21,37^ have been conducted to monitor the material ejection step independent of MALDI ions. For MALDI performed with laser spot sizes in the 100 µm-range (as is typical in classical applications such as proteomics and lipidomics), the number of ejected molecules was found to generally follow an exponential Arrhenius-type dependence on laser fluence *H*, once a detection threshold is surpassed. This relationship was shown to be directly linked to the desorption process described above^5,8,21^. Other experiments as well as simulations of the process, however, also indicated the occurrence of clusters in the plume and therefore point towards ablation like processes^22,25,28^.

When MALDI experiments are carried out with different laser spot sizes a number of researches have reported a peculiar spot size effect. They observed a particular increase in threshold fluence needed for the production of ions with decreasing spot size. This becomes especially apparent in spot sizes below ~20 µm which are typical for MALDI-imaging applications. Altogether studies on this phenomenon offer somewhat conflicting empirical models to describe this unexpected spot size effect^8,10,13,15,30^. While all approaches agree on an over proportional increase of threshold fluence with decreasing beam diameter, the underlying mechanism remains unclear. Utilizing laser postionization to probe neutrally desorbed molecules, Dreisewerd *et al*. observed a similar steep increase in laser fluence needed for material ejection, when focal spot sizes are decreased to the low ten to sub ten-micrometre diameter range^8^.

To obtain deeper insight into the spot size and fluence dependence of the material ejection step in MALDI – and in a broader sense into the interaction of nanosecond-pulsed UV-laser irradiation with surfaces of organic crystals – we here made use of two complementary methods. The first is based on our recently introduced “MALDI-2” postionization (PI) technique (see also Fig. S1A) and takes up similar approaches of earlier work^8,35,36,38,39^. With MALDI-2, a second pulsed UV-laser beam intercepts the expanding MALD(I) plume within a fine vacuum environment. In contrast to most of the earlier work the combination of collisional cooling and subsequent orthogonal time of flight (oTOF-MS) detection of the ions leads to an effective decoupling of ionization and detection. This way desorption conditions can be changed independently of mass spectrometric performance^39,40^. With the postionization confined to a small volume about 500 µm above the sample and triggered at a specific time predetermined by the delay between the two laser pulses, the intensity of the MALDI-2 ions can be used to probe the velocity distribution of ejected material at high temporal resolution.

The second employed method is based on the analysis of the compressive stress wave that is produced by the laser-induced ejection under ambient conditions (Fig. S1B)^21^. In good approximation, this photo-acoustic approach provides a near quantitative measure of the amount of material that is removed per laser pulse and, in this way, enables a more direct comparison with model considerations. Both sets of experiments were performed with focal dimensions of the primary laser beam ranging from ~5 to 200 µm by cross section, thereby covering both standard MALDI and emerging MALDI-MSI applications. To ensure well defined irradiation conditions, rectangular flat-top (FT) beam profiles were generated with a fundamental beam shaper^13^ (Fig. S1) and used throughout the presented experiments. Not only do they provide a homogeneous distribution of energy over the whole irradiated area, but also the steep flanks of the profile prevent a sizable enlargement of ablation craters with increasing laser pulse energy as observed with standard Gaussian beams. While complementary results acquired with Gaussian beams are found in the SI (Fig. S2) and lead to very similar results, the analysis of the influence of beam profile is not within the scope of this paper. For the experiments 2,5-Dihyroxybenzoic acid (DHB) and α-cyano-4-hydroxycinnamic acid (HCCA) were selected as classical, well-characterized MALDI matrices^5,41,42^.

## Results

### Plume Dynamics Probed by Laser Postionization

All postionization experiments were performed with sections of homogenized porcine brain and a sublimation/recrystallization protocol for DHB and HCCA matrices. These standardized samples and preparation conditions were established for fundamental studies in earlier work and are described there in greater detail^39^. Six different flat-top spot sizes ranging from 25 to 150 µm were investigated (7–100 µm for Gaussian beam profiles depicted in the SI). In line with previous studies in positive ion mode, potassiated lipids, mainly different PC species, form the largest peaks in the acquired MALDI mass spectra^39,43^ (Fig. S3). Also in line with earlier work, MALDI-2 spectra are dominated by protonated and radical matrix ions. In the lipids mass range, [M + H]^+^ ion species of various phospho- and glycolipids are the largest beneficiaries of the postionization with signal intensities increasing several orders of magnitude^39,43^. Signal intensity of potassiated and sodiated lipid ions remain largely unchanged (Fig. S3). Qualitatively, similar mass spectral profiles were obtained with all probed spot sizes. In line with the described spot size effect, laser fluence had to be increased about an order of magnitude between the largest and the smallest applied spot for optimized performance. Additionally notable differences in the extent of thermal fragmentation for different spot sizes were apparent that will be discussed in more detail below.

**Figure 1:**
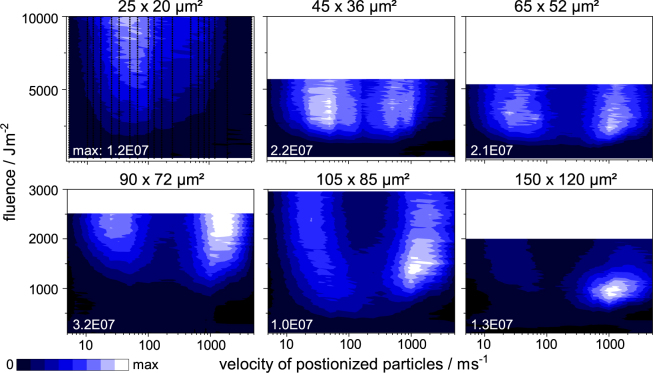
Total ion counts collected from sections of homogenized brain coated with DHB for different laser fluences and delay times. TIC intensity is displayed in false colour and plotted against employed laser fluence and particle velocity as obtained with laser postionization 500 µm above the sample. Black dots in the upper left subfigure indicate collected data points. Respective laser spot size and maximum of the obtained TIC are denoted in the subfigures.

Compared to our previous MALDI-2 work^39^, a lower N_2_ buffer gas pressure in the ion source of ~0.7, instead of 2–4 mbar was chosen. At this pressure only moderate modulating effects of the collision gas occur on the probed length scale resulting in velocities comparable to those reported for high vacuum conditions^29,30,44^. To first order the probed velocity distributions can therefore be assumed to widely reflect a “free” expansion into vacuum. Under the given conditions laser PI increases the total ion count (TIC) by 1–2 orders of magnitude as compared to normal MALDI measurements. Because the TIC is therefore largely dominated by ejected neutrals that were ionized ca. *Δz* = 500 µm above the ejection site at the time of the postionization pulse, it constitutes a suitable measure for plume dynamics. To visualize the effect of the focal spot size and laser fluence on this parameter, the TIC of standardized experimental conditions using tissue homogenate and DHB as a matrix is displayed in form of false color-coded heat maps in Fig. 1. To facilitate data interpretation, instead of the delay *τ* between the two laser pulses, the mean velocity of the postionized particles, *v*_*m*_ = *Δz/τ*, is plotted along the abscissa. Black dots, representatively plotted in the upper left heat map, denote the acquired data points. Equivalent data on HCCA is presented in Fig. S4.

Similar to results by Dreisewerd *et al*., in the dimension of fluence, the spot size effect is affirmed also for the total amount of postionized material^8^. While fluences around 10000 Jm^−^² are needed to produce maximum TIC values for the smallest spot of 25 × 20 µm² (and even 150000 Jm^-^² for the smallest Gaussian spot of 7 × 5 µm², see SI), values of only about 1000 Jm^-^² suffice for a laser spot of 150 × 120 µm². Next to this fluence dependence, also dependencies of the velocity become apparent in Fig. 1 and reveal that most molecules are found in two distinct velocity regimes. For the smallest probed spot, the expansion dynamics are clearly dominated by a slow plume component with a mean velocity of a few 10 m/s produced at high fluences just below 10000 Jm^−2^. Upon a step-wise increase of laser spot size, in an intermediate region between 45 × 36 µm² and 105 × 85 µm², a second region of intense TIC appears at velocities of 1000–1500 m/s. With further increase of spot size this regime gains in intensity while its maximum is produced at continuously lower fluence values. At the largest spot size of 150 × 120 µm² most of the postionized material is found in this fast component produced in a narrow fluence band around 1000 Jm^−2^. The decrease of the TIC at fluences higher than the respective maximum can tentatively be attributed to an increased fragmentation of matrix molecules upon desorption. This may induce a shift of absorptivity in the fragments or a change in chemical characteristics and thereby prevents effective resonant postionization.

The apparent split in the velocity distribution can be interpreted as two distinctively different material ejection regimes intertwining for typical MALDI conditions. The fast regime is observed at velocities comparable to those measured for molecular ion species in MALDI reported in the literature^29,44^. In particular registered velocities and their distribution are in good agreement with considerations for thermal desorption by Dreisewerd^5^. Also reporting similar velocities, Spengler and Kirsch suggest a plume expansion in the form of a non-ideal molecular jet induced by the process^30^. The second, slow modality, mainly observed for smaller spot sizes gains in intensity when distinctively higher fluences are deployed. Taking its slower velocity into account, and assuming similar kinetic energies for the ejected material, the mass of the ejecta calculates to about 100–4000 times the mass of the fast molecules. This assumption is corroborated by a similar cluster size calculated to about 1000 matrix molecules that was found experimentally by Musapelo and Murray using a differential mobility analyser as well as in simulations of the MALDI event in the ablation regime by Zhigilei^28,34,45^. Together the new and previously reported observations can be interpreted that the slow component is in fact an ejection of larger clusters from deeper layers carrying kinetic energies similar to those of single molecules ejected from the surface.

All together material ejection under MALDI conditions that leads to free molecules accessible to post ionization seems to be dominated by quasi-molecular desorption at large spots produced at moderate fluences and ablation-like process at small irradiated areas necessitating increased fluence values. Both mechanisms are triggered in a transition region of spot sizes between 30 and 100 µm for DHB and about 10 and 50 µm for HCCA (also see Fig. S4) where both velocity regimes are present in the collected data. While a dual modality in the velocity distribution of MALDI plumes has been described before^25,27,30,46^ here its characteristics can be linked to certain spot sizes and fluence regimes for the first time.

### Plume Temperatures

**Figure 2:**
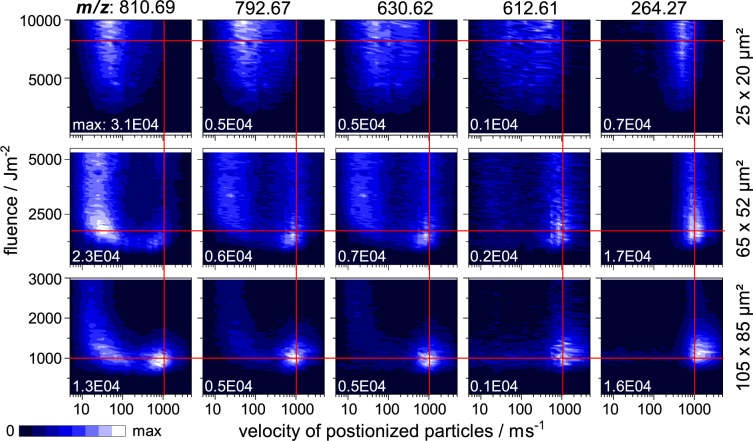
Signal intensity of [GalCer(d18:1/C24:1) +H]^+^ and five of its most prominent thermal fragments plotted against the employed fluence and particle velocity for three different spot sizes. Average activation energies needed for the production of the fragments increase from left to right (see Fig. S5 for details). Red lines denote fixed velocities and fluences to help with comparison between subfigures.

Thermal excitation by the MALDI-process and thereby the temperature of the early plume constitutes a key influence in the production of intact molecular ions, especially for weakly bound analyte species. The spot size and fluence dependence of this analytically important parameter can be studied by evaluating the signal intensities of intact galactosylceramide GalCer(d18:1/24:1) as well as a set of its characteristic thermal fragments all found in the spectra of the probed tissue homogenate. As determined by means of low-energy collisional-induced dissociation (CID) tandem mass spectrometry, each fragment is produced by gas-phase fragmentation as a result of distinct activation energies (Fig. S5). Therefore signal intensities of these fragments can be used as indicators of the internal energy transferred during material ejection and thereby probe the temperature in the early plume. Figure 2 shows the fluence-velocity distributions for the intact GalCer as well as its four fragments for three different spot sizes.

As revealed in the top row, for small spot sizes the energy transfer is largely independent of laser fluence and generally less prominent compared to larger spots. The intensity of all fragments slowly increases with fluence similarly to that of their precursor. While the intact analyte shows the highest intensity in the slow velocity domain around 50 m/s, the fragments seem to travel distinctively faster, revealing a correlation between thermal excitation and particle velocity. Signal intensity of the energetically most demanding fragment m/z 264.27 for example peaks just below 1000 m/s. It may be speculated that faster and/or smaller components of the plume produced by ablation carry a higher thermal load leading to increased fragmentation of analyte during their decay, while slower and larger clusters somehow “protect” the analyte from excessive heating.

With increasing spot size (middle and bottom row), intact analyte is also detected in the fast velocity regime produced at considerably lower fluences. In this domain the thermal load shows a clear dependence on both fluence and velocity. More energy demanding fragments are predominantly found at higher fluences and velocities, pointing to an increased transfer of kinetic as well as thermal energy to the analyte under these conditions. The observations made for this faster modality can be linked to a thermal desorption process occurring at larger spot sizes. Here material ejection is initiated by a high temperature in the upmost layers of the sample that is directly linked to the employed fluence. In this case, not only the detached particles gain velocity with increasing fluence, but also the temperature of surface and early plume shift to higher values. Consequently the amounts of energy transferred to intramolecular bonds in matrix and analyte molecules increases and leads to the observed fragmentation. Coherent with the findings in the first paragraph, the observations made for the plume temperature thereby support the idea of two distinctively different regimes of material ejection with distinctively different analyte fragmentation – thermal desorption from the surface and ablation of clusters from the bulk.

### Material Ejection Probed by Photoacoustic Experiments

The yield of the MALDI-2-process is selective on physicochemical properties of the ejected material and their interplay with wavelength and intensity of the PI-laser pulse as well as charge transfer reactions within the plume, all of which may vary with changes to the initial process of material ejection. Therefore registered ion signal intensities cannot straightforwardly be translated to quantitative amounts of ejected material. Consequently, some restraint has to be exercised in interpreting the MALDI-2-derived ion signals in a quantitative manner. We therefore made use of a complementary photoacoustic approach. Under ambient conditions all material that is ejected into the surrounding air during a MALDI event contributes to a pressure wave. This wave can be picked up by a microphone and its amplitude serves as a measure for the amount of ejected material^21^. In unimolecular desorption (or sublimation) all ejected material is directly transferred from solid to the gas-phase. Consequently the pressure wave is directly proportional to the amount of material removed from the surface. In the ablation of larger clusters the process is less straightforward as some of the material stays solid and may contribute less to the pressure wave. The total amount of material may therefore be underestimated. Considering a fast secondary uni- or oligomolecular decay of most of the emitted clusters^7,34^, however, a sizeable acoustic signal proportional to the amount of material ejected during ablation can be expected. Because of the high back pressure, plume development is largely suppressed for both mechanisms and only the initial process of material removal can be probed.

**Figure 3:**
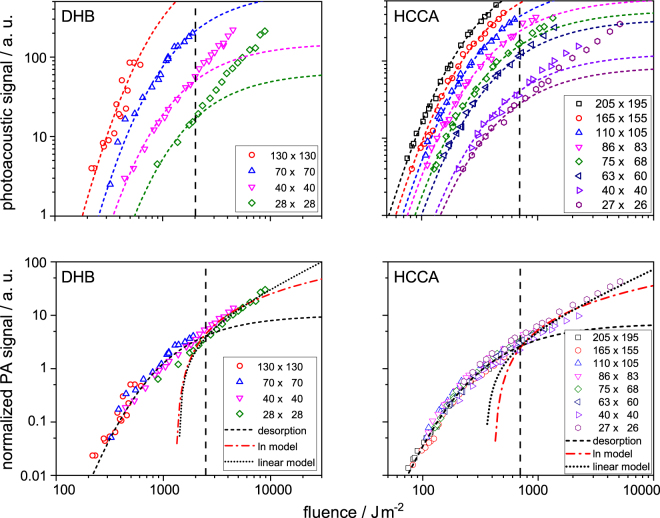
MALDI material ejection step as probed by photoacoustic analysis. PA signals were recorded as a function of laser fluence and rectangular flat top spot size from microcrystalline preparations of neat DHB and HCCA matrices (upper graphs). The bottom graphs display the same data set after normalization to the focal spot size, *A*. The coloured dashed lines depict a best fit of the quasi-thermal desorption model of Eq. (1) to the data, the red dash-dotted lines a best fit of the ln model according to Eq. (2), and the black dotted lines a fit to the linear model of Eq. (3). All data points are mean values of 50 laser shots with a standard deviation of 5–10%.

Figure 3 shows the data recorded on a layer of DHB and HCCA sublimated onto a transparent CaF_2_ target (see methods for details). Tests with sections of matrix-coated brain tissue homogenates as used in the MALDI-2 experiments did not result in a discernibly different response. The upper graphs show the photoacoustic signal intensities for different laser spot sizes (equivalent results with Gaussian beam profile are presented in Fig. S6). As expected, photo-acoustic response decreases for smaller spots since less material is ejected from smaller volumes. To compare all employed spot sizes, the PA signal was normalized to the irradiated area and is depicted in the bottom row of Fig. 3. Remarkably all normalized data points fall on one line. This indicates that the amount of material ejected from a defined unit area irradiated by the laser only depends on the energy deposited upon that area and is independent of the spot size employed.

The quantitative character of the data now allows for the evaluation of different mathematical models proposed for the description of material ejection for the probed fluences. Building on the idea of two distinctively different regimes of material ejection derived from the PI results, a thermal desorption/sublimation based model is expected to describe the low fluence region while a volume ablation should dominate the high fluence regime. For thermal desorption, an exponential Arrhenius-type dependence of the material ejection *N*_*des*_ on the lattice temperature near the surface is predicted. This temperature is in turn directly proportional to the applied laser fluence *H* with an energy transformation factor *η*, proportional to the absorption coefficient of the matrix at the given wavelength. This leads to a description of the form^5,8,47^:

1 $${N}_{{des}}\,\propto \,\exp (\frac{{-E}_{a}}{{k}_{b}({T}_{{0}}+\eta H)})$$

Where *E*_*a*_ is the activation energy to disintegrate the matrix crystal lattice, *k*_*b*_ is the Boltzmann constant and *T*_0_ the initial sample temperature. For fluences below a transition threshold of about 2000 Jm^−2^ for DHB and 700 Jm^−2^ for HCCA the collected data is well described by this model. A best fit using *P* and *η* as fitting parameters and *E*_*a*_ set to 0.5 eV (in line with values reported earlier^8,21,22^, see SI for details) resulted in the coloured dashed lines presented in Fig. 3. Evidently the model is well suited to describe material ejection in the tested fluence regime. Fits to the model of similar quality were found for both investigated matrices (DHB and HCCA) and also for Gaussian beam profiles (see Figs S4 and S5 and Tables S1 and S2 for fitting parameters). It is also worthwhile to consider the values retrieved for the fit parameter *η*. As it describes the conversion of laser fluence to thermal energy it is directly dependent on matrix absorptivity^9,12,21,48^. Fittingly the difference of reported absorption values for the matrices DHB and HCCA at the employed wavelength of about a factor 5 is reasonably well retrieved from the fit with a value of about 3.5^49^.

At elevated fluences above the transition threshold data points increasingly deviate to values larger than predicted by the desorption model and disclose a change in the material ejection processes. Following the ideas proposed earlier, in this high fluence regime ablation from the bulk could be the dominating process. Still under lively discussion, the exact mechanisms of laser ablation of solids are dependent on a highly complex interplay of physical and chemical properties of the substrate and irradiation parameters like fluence, pulse duration and wavelength^33,50–52^. For typical MALDI conditions with ns-pulses hitting molecular solids, mechanisms like phase explosion, tensile fragmentation, as well as melting and boiling have been proposed. The share of each mechanism on the overall process for a given MALDI-experiment however is highly speculative and may vary greatly between setups and irradiation parameters. Consequently mathematical models describing material ejection by ablation as a function of fluence have to be greatly simplified. The simplest model for ablation, here called *linear model*, proposed to the context of MALDI by Rohlfing *et al*.^23^ follows the form

2 $${N}_{{abl}}\,\propto \,{H}-{H}_{thr}$$

Here *H*_*thr*_ is inversely proportional to the absorptivity and describes a threshold fluence sufficient to heat the irradiated volume to a threshold temperature necessary for ablation. Like in classical boiling all excess energy provided to the heated volume above this temperature is directly proportional to the amount of ejected material regardless of the energy distribution within the volume.

The second discussed model, here called *ln model* is based on a threshold energy density *E*_*V*_ for ablation^5,47^. Here material is ejected if the energy density initially deposited by the laser pulse surpasses *E*_*V*_ in a given volume element. With an exponential decay of the laser intensity entering an absorptive medium, this defines a critical depth down to which ablation occurs. Thereby the amount of ablated material ${N}_{{\rm{abl}}}$, is described by

3 $${N}_{{abl}}\propto {\mathrm{ln}}^{n}(\frac{H}{{H}_{thr}})\,{\rm{for}}\,H\, > {H}_{thr}$$

where *n* factors in the laser beam profile (between 1 for a flat-top and 2 for a Gaussian beam profile). Again *H*_*thr*_ is inversely proportional to the absorptivity.

The red dash-dotted *(ln model*) and black dotted lines (*linear model*) in Fig. 3 and Fig. S6 represent best fits of both discussed models to the data above the transition fluence. Both models are well suited to explain the experimental findings by an onset of ablation processes at elevated fluences. While the situation close to the transition is well represented by both models, the *linear model* delivers better fits for high fluence values. Taken with advisable precaution, this may point to a correlation of ablated material with the total amount of deposited energy rather than its initial spatial distribution.

## Discussion

Both complementary datasets suggest that the process of material ejection in MALDI involves at least two distinctively different mechanisms. At low fluences a thermally driven uni- or oligomolecular desorption/sublimation from the upmost layers of the sample is dominant. Higher fluences initiate volume processes - for lack of deeper understanding pooled under the term ablation – that produce larger clusters and particles. The two datasets however also seemingly display dissent on the spot size dependence. On the one hand the normalized PA data shows that in general the amount of material ejection from a certain area as well as the underlying mechanism is only dependent on fluence and largely independent of the applied spot size. On the other hand postionization data suggests that the optimal fluence region to produce molecules accessible to MALDI-2 drastically changes with spot size. For large spots a narrow window in the low fluence region leads to the most intense signal while for smaller spots much higher fluences are needed for optimal MALDI-2 results. On closer examination these findings suggest an involvement of plume development after initial ejection that effects the accessibility of molecules to successful postionization. Together this hints towards an involvement of the thermodynamic evolution of the plume to cause of the spot size effect in MALDI-2 and most probably also for classical MALDI. These ideas are corroborated by experimental findings. For the largest spot the fluence window for optimal postionization and also classical MALDI (roughly 750 to 1300 Jm^−2^ for DHB) lays well within the desorption region with little to no contribution from ablation. As predicted by the model this regime is dominated by fast molecules (Fig. 1). For the smallest spot the optimal fluence window is positioned in a region with dominant ablation (>8500 Jm^−2^), again corroborated by the velocity distribution showing mostly slow projectiles. All spot sizes between the two extremes have optimal fluences in a transition region with both mechanisms contributing and their velocity distributions concurrently show both fast and slow components. This leads to the conclusion that while the starting conditions of the plume are governed by the mechanism of material ejection and thereby only by fluence, the desired outcome of gaseous but intact analyte as well as matrix molecules needed for MALDI-2 is largely dependent on thermodynamic development inside the evolving plume, that is, in turn, influenced by the employed spot size.

**Figure 4:**
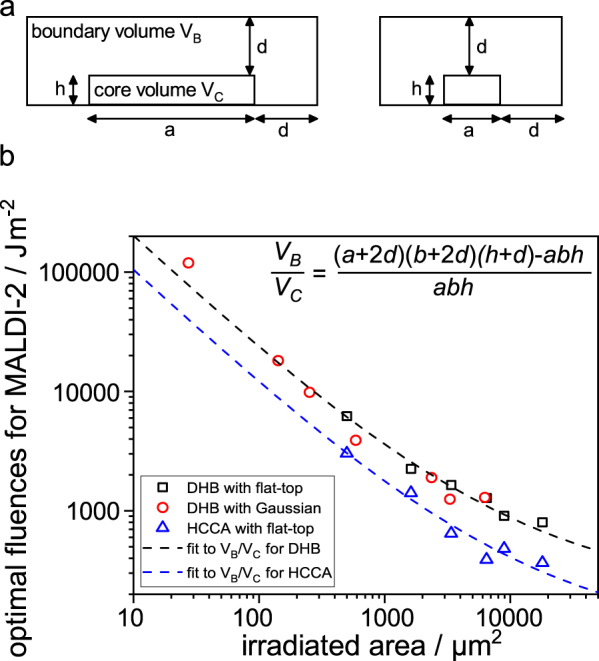
(**a**) Schematic representation of core volume *V*_*C*_ and boundary volume *V*_*B*_ for a large (left) and small (right) laser spot size. *a*, *b*: edge lengths of rectangular laser spot; *h*: height of early laser plume; *d*: thickness of boundary layer around expanding plume. (**b**) Optimal laser fluence for MALDI-2 with DHB and HCCA plotted against the irradiated area (spot size) *A*. Blue and red dashed lines represent best fits of Eq. (4) for both matrices. (See SI for details on the fitting procedure)

The temperature, pressure and density and their development in time and space inside the plume largely govern the distribution of internal energy after initial material ejection and thereby determine the fate of ejected molecules. Cold or very dense conditions may lead to an incomplete break up of clusters or condensation of molecules to larger chunks^52^. On the contrary elevated temperatures lead to fragmentation of analyte and matrix as discussed with Fig. 2. Therefore the assumption seems reasonable that only a narrow passage through the phase-diagram during plume development leads to the desired outcome. While the initial process of material ejection is preset only by the employed fluence, the thermodynamic environment during the subsequent early expansion of the plume is largely influenced by inertial confinement of the expanding material^33^. Next to the amount of initial energy introduced to the volume by the laser pulse, boundary conditions therefore largely influence the expansion of the plume and its confinement. In a boundary region around the expanding front, large temperature and pressure gradients lead to a fast redistribution of thermal and kinetic energy away from the core of the plume^33^. Further away from the boundary, thermodynamic conditions are more spatially homogenous and less energy scrambling can appear. Consequently the overall thermodynamic state of the early plume is dependent on the relation of a boundary volume V_B_ and a core volume V_C_ (Fig. 4). For a small spot the ratio is high and a larger fraction of energy provided by the initial laser pulse can be displaced leading to a more efficient cooling and thinning of the plume. For large spots the ratio declines and a larger fraction of energy stays within the plume. The core volume stays hotter and denser. Based on an assumed constant thickness *d* of the boundary region, a height *h* of the evolving plume and a rectangular irradiated area *A* = *a* * *b* the ratio V_B_/V_C_ can in a first approximation be expressed as shown in Fig. 4b. Following the presented line of argument this ratio should now be proportional to the energy loss from the evolving plume due to boundary effects. Consequently this energy loss has to be compensated by the energy delivered by the laser pulse and fluence has to be adapted to the spot size in order to achieve favourable thermodynamic conditions. To test this hypothesis V_B_/V_C_ can be compared to the fluence needed to reach these optimal conditions as a function of spot size.

As shown by a number of publications the interpretation of a fluence dependent ion signal intensity *I(H)* in MALDI and MALDI-2 to evaluate optimal conditions is complicated^8–10,13,15,21^. In most cases the increase of ion signal intensity with fluence (*H*) is counteracted by fragmentation processes leading to characteristic S-curves. Building on recent work by Robinson *et al*., however, the maximum slope *R = dI(H)/dH* of the relation and the respective fluence *H*_*R*_ is suitable to describe these optimal conditions^49^. Figure 4 shows *H*_*R*_ derived from the TIC of MALDI-2 data for both employed matrices plotted against the applied spot size *A*. Best fits to Eq. (4) reveal a good fit to the model (see SI for details). These results affirm a possible involvement of the boundary region of the evolving plume in the spot size effect. For both matrices the thickness of the evolving plume *h* was set to 20 µm and the width of the boundary region *d* was calculated to ~40 µm, a reasonable value considering the employed spot sizes. Similar values for *d* were achieved when *h* was varied over three orders of magnitude (see SI for details).

In summary all data points to the conclusion that mechanism and amount of ejected material are independent of the spot size. Low fluence values lead to quasithermal desorption and additional ablation sets in at fluences beyond a transition threshold. The production of intact molecules accessible to postionization within the laser plume however underlies a spot size effect and is only observed under certain favourable thermodynamic conditions. These conditions depend on the energy distribution within the evolving plume. We propose that energy scrambling, critical to the thermodynamic development after the initial ejection, is governed by the volume ratio of a boundary layer to the core of the plume. This leads to a spot size dependent energy flux out of the core volume that needs to be compensated by an adapted fluence in order to produce optimal signal intensities in MALDI-2. Care has to be taken when translating these results to classical MALDI. Here next to mere material ejection, ionization takes place within the early plume as well and might favor slightly different thermodynamic conditions. It is however reasonable to conclude that similar scrambling processes of thermal and kinetic energy during plume development play key roles in the spot size effect in classical MALDI as well.

## Materials and Methods

### Sample preparation

Porcine brain was purchased at a local butcher shop and processed as described elsewhere^36^. 15 µm-thick sections of porcine brain homogenate were coated with DHB or HCCA matrix (both from Sigma-Aldrich, Steinheim, Germany) using one of two typical MALDI preparation protocols that were described in detail elsewhere^13,39^. A pneumatic spraying system (HCCA, photoacoustic) or a sublimation/recrystallization protocol (all other) lead in both cases to crystal sizes smaller 10 µm.

### Laser Beam Shaping/Steering and Online-Control of focal beam profiles

The set-up for beam shaping, steering, and online-control of focal intensity for the primary MALDI laser was described in detail previously^13^. Briefly, diode-pumped solid state lasers with Gaussian beam profiles (M² < 1.15) were employed. The beam was manipulated to a square flat-top profile by a fundamental beam shaper (FBS2, TOPAG, Darmstadt, Germany). Laser spot size was varied from ~5 to 200 µm diameter/edge length by telescope optics before final focusing. The beam profile was either monitored online with a beam profiler (SP620U, Ophir Spiricon, Darmstadt, Germany) for the photoacoustic setup or simply optically by means of the ablation craters (postionization experiments).

### Postionization Experiments

The set-up for the MALDI-2 experiments is sketched in Fig. S1A and described in more detail elsewhere^39^. Briefly, a frequency-tripled Nd:YLF laser (Explorer, Spectra-Physics, Santa Clara, CA) of 349 nm wavelength and 7 ns pulse duration was used as the primary MALDI laser with a shot-to-shot pulse energy stability of <3%. The MS analysis was achieved by use of a QTOF-type Synapt G2-S mass spectrometer (Waters, Manchester, UK) with a modified MALDI ion source^39,53^. For postionization an optical parametric oscillator laser (OPO, versaScan with uvScan for SHG generation, GWU-Lasertechnik, Erftstadt, Germany) with 6 ns pulse duration was tuned to 280 nm. The beam was steered and focused to produce a ~100 µm-wide beam waist propagating at an approximate distance Δz of ~500 µm centrally above the focal MALDI laser spot. The pulse-to-pulse energy stability of the OPO laser was about 5%. Both lasers were operated at 20 Hz and synchronized by use of a custom-made delay generator. The jitter of both laser lead to an error of about ±50 ns, while the delay was varied from 100 ns (postionizing particles with velocity v = Δz/τ = 5000 m/s) to 100 µs (5 m/s). All mass spectra (data point) for a particular spot size/fluence/delay combination were recorded over 20 laser shots. To simplify data acquisition, during one experimental run (given spot size and delay), the fluence was automatically linearly increased using custom-made software and a neutral density gradient filter, while the sample stage was continuously moved to only irradiate fresh sample areas. The reported fluence values are therefore averages of a small fluence increment (±a few 10 to 100 J/m² - dependent on the fluence range covered). To account for the sizable, but fairly constant chemical background as produced by the PI laser from residual gas constituents, all evaluated mass spectra were background-subtracted. Data visualization in the form of heat maps was done with the software Origin (OriginPro 2016G, OriginLab, Northampton, MA).

### Photoacoustic Experiments

The set-up for the photoacoustic experiments is sketched in Fig. S1B and is described in reference^21^. Material ejection was induced with a frequency-tripled Nd:YAG laser (diodescope, Bioptic Lasersysteme, Berlin, Germany). The minor difference in emission wavelength (355 *vs*. 349 nm), laser pulse duration (20 *vs*. 7 ns), as compared to the Nd:YLF laser used in the MALDI-2 experiments, and the difference in angles of incidence (90° *vs*. 45°) is assumed to not have any relevant effects^11,48,54^. Samples were mounted inside a sealed quartz cuvette and moved using a contact-free magnetic system (Fig. S1B). Fresh sample spots were irradiated at each laser pulse. A repetition rate of 1 Hz was used with maximum pulse energy of ~40 µJ. Data evaluation was performed as described.

### Data availability

All datasets generated and analysed during this study are available from the corresponding author on reasonable request.

## Electronic supplementary material

Supporting Information
