# Supplementary material for: New insights into mechanisms of material ejection in MALDI mass spectrometry for a wide range of spot sizes
Source: Sci Rep. 2018 May 17;8:7755. doi: 10.1038/s41598-018-25946-z (PMC5958139; doi:10.1038/s41598-018-25946-z)
Supplement: Supplementary file 1 — Supporting Information [file 41598_2018_25946_MOESM1_ESM.docx]

**Supporting Information for**

**New insights into mechanisms of material ejection in MALDI mass spectrometry for a wide range of spot sizes**

Marcel Niehaus^1^, Jens Soltwisch^1,2,*^

^1^ Institute for Hygiene, University of Münster, Robert-Koch-Strasse 41, 48149 Münster, Germany

^2^ Interdisciplinary Center for Clinical Research (IZKF), University of Münster, Domagkstrasse 3, 48149 Münster, Germany

Key words: MALDI mechanisms, MALDI-2, Material ejection, Spot size, Photo acoustics

^*^ Correspondence to: Jens Soltwisch ([jenssol@uni-muenster.de](mailto:jenssol@uni-muenster.de))

**Table of contents**

Figures S1 – S6

Table S1 - S2

Supplementary Information for material and methods

Fig. S1: Schematic of the setups used for postionization (a) and photoacoustic (b) experiments.

Fig. S2: MALDI plume dynamics as probed by laser postionization (MALDI-2) with the unaltered Gaussian beam. Heat maps visualizing the total ion counts recorded from DHB-coated homogenized brain tissue sections as a function of laser fluence and mean particle velocity, *v_m_=Δz/τ*, where *Δz* = 500 µm is the distance of the postionization beam from the sample, and *τ* is the delay between the two laser pulses. Numbers in the bottom left corner denote the maximum ion counts. Black dots depict the collected data points.

Fig. S3: Mass spectra of normal MALDI (red traces; bottom) and the postionization experiments (black traces; top; MALDI-2) for three different spot sizes and delay times optimized for high ionization efficiency. The postionization spectra are dominated by matrix ions (m), but many analytes species (lipids) are ionized by secondary gas phase reactions as already reported ^1^

.

Fig. S4: MALDI plume dynamics as probed by laser postionization (MALDI-2). Heat maps visualizing the total ion counts recorded from HCCA-coated homogenized brain tissue sections as a function of flat-top spot size, laser fluence and mean particle velocity, *v_m_=Δz/τ*, where *Δz*= 500 µm is the distance of the postionization beam from the sample, and *τ* is the delay between the two laser pulses. Numbers in the bottom left corner denote the maximum ion counts. The vertical black dots depict the experimental data points.

Fig. S5: Signal intensities of [GalCer(d18:1/C24:1)+H]^+^ (m/z= 810.69) and selected CID-fragments in dependence of the applied activation energy (left) and the chemical structure and fragmentation pathways of the molecule (right). Fragments with *m/z* 792.67 and 612.61 represent loss of H_2_O from the precursor and fragment *m/z* 630.62 respectively.

Fig. S6: MALDI material ejection step as probed by photoacoustic analysis. PA signals were recorded as a function of laser fluence and Gaussian focal spot size from microcrystalline preparations of neat DHB and HCCA matrices (upper graphs). The bottom graphs display the same data set after normalization to the focal spot size, *A*. The coloured dashed lines depict the best fit of the quasi-thermal desorption model of Eq. (1) to the data, the red dash-dotted lines a best fit of an ablation model according to Eq. (2), and the black dotted lines a fit to the linear model of Eq. (3). All data points are mean values of 50 laser shots with a standard deviation of 5-10%.

Table S1: Fit values for desorption, ln and linear model as displayed in Fig. 3 following equations I, II and III for the flat-top spot sizes. For all fits the parameter *E_a_* of the desorption model was set to 0.5 eV to enable better comparison between the other parameters (see text for further explanations).

| Spot size / µm² | | desorption | | ablation | | linear | |
| --- | --- | --- | --- | --- | --- | --- | --- |
|  | P* | | η / K m^2^ J^-1^ | K* | H_thr, abl_ / J m^-2^ | F* | H_thr, lin_ / J m^-2^ |
| DHB |  | |  |  |  |  |  |
| 130 x 130 | 2500 | | 2.5 |  |  |  |  |
| 70 x 70 | 675 | | 2.5 |  |  |  |  |
| 40 x 40 | 150 | | 2.5 |  |  |  |  |
| 28 x 28 | 65 | | 2.0 |  |  |  |  |
| normalized | 10 | | 2.5 | 40 | 1500 | 0.0035 | 1400 |
|  |  | |  |  |  |  |  |
| HCCA |  | |  |  |  |  |  |
| 205 x 195 | 2040 | | 9.0 |  |  |  |  |
| 165 x 155 | 1415 | | 8.5 |  |  |  |  |
| 110 x 105 | 1010 | | 8.0 |  |  |  |  |
| 86 x 83 | 650 | | 8.0 |  |  |  |  |
| 75 x 68 | 450 | | 7.5 |  |  |  |  |
| 63 x 60 | 350 | | 7.0 |  |  |  |  |
| 40 x 40 | 125 | | 7.0 |  |  |  |  |
| 27 x 26 | 85 | | 7.0 |  |  |  |  |
| normalized | 7 | | 8.0 | 22 | 410 | 0.0075 | 650 |

*Empirical proportionality factors

Table S 2: Fit values for desorption, ablation and linear model as displayed in Fig. S6 following equations I, II and III for the Gaussian spot sizes. For all fits the parameter *E_a_* of the desorption model was set to 0.5 eV to enable better comparison between the other parameters (see text for further explanations).

| Spot size / µm² | | desorption | | ablation | | linear | |
| --- | --- | --- | --- | --- | --- | --- | --- |
|  | P* | | η / K m^2^ J^-1^ | K* | H_thr, abl_ / J m^-2^ | F* | H_thr, lin_ / J m^-2^ |
| DHB |  | |  |  |  |  |  |
| 130 x 120 | 500 | | 3.0 |  |  |  |  |
| 95 x 95 | 400 | | 3.0 |  |  |  |  |
| 50 x 50 | 170 | | 3.0 |  |  |  |  |
| 30 x 30 | 40 | | 2.5 |  |  |  |  |
| 23 x 23 | 12 | | 2.5 |  |  |  |  |
| 16 x 18 | 8 | | 2.5 |  |  |  |  |
| 8 x 9 | 2 | | 2.5 |  |  |  |  |
| normalized | 5 | | 2.5 | 40 | 1500 | 0.003 | 1000 |
|  |  | |  |  |  |  |  |
| HCCA |  | |  |  |  |  |  |
| 190 x 200 | 450 | | 13.0 |  |  |  |  |
| 140 x 150 | 350 | | 12.0 |  |  |  |  |
| 100 x 110 | 250 | | 13.0 |  |  |  |  |
| 75 x 85 | 110 | | 12.0 |  |  |  |  |
| 60 x 61 | 55 | | 10.0 |  |  |  |  |
| 40 x 45 | 15 | | 11.0 |  |  |  |  |
| 23 x 22 | 7 | | 12.0 |  |  |  |  |
| normalized | 2 | | 12.0 | 12 | 400 | 0.004 | 200 |

*Empirical proportionality factors

**Fitting procedure for photoacoustic data**

In a first step, all data sets (all spot sizes for both beam profiles and matrices) were fitted to the desorption model (Eq. 1) independently with *P*, *E_a_* and *η* as fitting parameters using gnuplot (gnuplot 4.6; www.gnuplot.info). Only the data sets obtained with larger spot sizes resulted in good quality fits with similar parameter values. The values generated for *E_a_* were in the range of 0.47 eV to 0.52 eV in line with several results in the literature ranging from 0.37 to 0.7 eV ^2–4^ for both matrices. Therefore a value fixed to 0.5 eV was chosen for a second fitting process to reduce fitting uncertainties of the other parameters. All fitting parameters are displayed in Table S1. Data sets generated with smaller laser spot sizes are described well by the desorption model only up to a specific fluence. To determine the threshold fluence, the fitting range was gradually expanded to higher values until results for the parameter η became unstable. Above the threshold fluence, data could not be comprehensively described by the model for desorption. Experimental values were deviating to higher values, as visible in Fig. 3 for both the not normalized and normalized data. For fluences higher than threshold, normalized data was fitted to the ln (Eq. 2) or linear model (Eq. 3) with reasonable results.

**Energy dissipation via a boundary volume of the evolving MALDI plume**

In order to obtain a consistent measure for mass spectrometric performance, the optimal laser fluence *H_R_* for MALDI-2 for all spot sizes was determined according to Robinson *et al*. ^5^. It is defined as the fluence at the point of maximum slope of ion intensity *I* as a function of fluence *H* as determined by d*I*(*H*)/d*H* = max. For differentiation data was smoothed with a moving average of 5. Please note that this procedure works well for larger spot sizes above 50 µm and is less reliable for smaller spot sizes. Here the S-curve is less pronounced, because the behaviour of the ion count differs distinctly due to the effects explained in the main manuscript (e. g. less fragmentation with smaller spot sizes). Nonetheless a less pronounced maximum slope was detected for the smaller spot sizes and the *H_R_*.

For the ion count the integrated ion signal over the measured range of velocities was used to account for differences in velocity distributions with varying spot size. Differentiation and integration of the data was performed with the software Origin (OriginPro 2016G, OriginLab, Northampton, MA).

*Fitting procedure*

The ratio of the boundary region *V_B_* and the inner core volume *V_C_* as shown in the main manuscript,

$$\frac{V_{B}}{V_{C}}= \frac{\left( a+2d \right)\cdot\left( b+2d \right)\cdot\left( h+d \right)-abh}{abh},$$

can be simplified and expressed in dependence of the area *A* of the rectangular laser spot with edge lengths of *a*, *b*. With

$$A=a\cdot b$$

and the fixed ratio *r* of the two edge lengths

$$r=\frac{a}{b}$$

this leads to:

$\frac{V_{B}}{V_{C}}=\frac{1}{A}\left( \frac{4d^{3}}{h}+4d^{2} \right)+\frac{1}{\sqrt{A}}\cdot\frac{\left( r+1 \right)}{\sqrt{r}}\cdot\left( \frac{2d^{2}}{h}+2d \right)+\frac{d}{h}$ (Eq. 4)

Here *d* represents the thickness of the boundary region where energy can dissipate and *h* is the height of the ejected particle cloud (see main manuscript).

The optimal laser fluence *H_R_* can now be linked to this volume ratio with a proportionality factor *k*:

$$H_{R}=k \cdot\frac{V_{B}}{V_{C}}.$$

The fitting process was performed with a value of *r*=0.8 as approximated ratio between the two edge lengths of the rectangular flat-top profile as indicated by the angle of incidence of the laser beam. The remaining unknowns besides the fitting parameter *d* for the thickness of the boundary volume are a proportionality factor *k* and the height *h* of the inner core volume. The later value is ultimately not known since it is a parameter of the evolution of the MALDI plume in time and space. The fit was therefore performed for values for *h* in between the approximated laser penetration depth of 200 nm and a three orders of magnitude larger plume in the gas phase of 200 µm to account for a large set of parameters. In this wide span the retrieved value of the fit parameter *d* changed only slightly from 43.6 µm (*h*=0.2 µm) to 27.9 µm (*h*=200 µm) for DHB (with an according change to the other fit parameter *k*).

References for Supporting Information

1. Soltwisch, J. *et al.* Mass spectrometry imaging with laser-induced postionization. *Science* **348,** 211–215 (2015).

2. Soltwisch, J., Jaskolla, T. W. & Dreisewerd, K. Color Matters—Material Ejection and Ion Yields in UV-MALDI Mass Spectrometry as a Function of Laser Wavelength and Laser Fluence. *J. Am. Soc. Mass Spectrom.* **24,** 1477–1488 (2013).

3. Dreisewerd, K., Schürenberg, M., Karas, M. & Hillenkamp, F. Influence of the Laser Intensity and Spot Size on the Desorption of Molecules and Ions in Matrix-Assisted Laser-Desorption Ionization with a Uniform Beam Profile. *Int. J. Mass Spectrom. Ion Process.* **141,** 127–148 (1995).

4. Zhigilei, L. V., Leveugle, E., Garrison, B. J., Yingling, Y. G. & Zeifman, M. I. Computer Simulations of Laser Ablation of Molecular Substrates. *Chem. Rev.* **103,** 321–348 (2003).

5. Robinson, K. N., Steven, R. T. & Bunch, J. Matrix Optical Absorption in UV-MALDI MS. *J. Am. Soc. Mass Spectrom.* **29,** 501-511 (2017).
